# Supplementary material for: Single-color, ratiometric biosensors for detecting signaling activities in live cells
Source: eLife. 2018 Jul 3;7:e35458. doi: 10.7554/eLife.35458 (PMC6037473; doi:10.7554/eLife.35458)
Supplement: Transparent reporting form [file elife-35458-transrepform.docx]

***eLife’s* transparent reporting form**

We encourage authors to provide detailed information *within their submission* to facilitate the interpretation and replication of experiments. If you have any questions, please contact us: [editorial@elifesciences.org](mailto:editorial@elifesciences.org).

**Sample-size estimation**

- You should state whether an appropriate sample size was computed when the study was being designed
- You should state the statistical method of sample size computation and any required assumptions
- If no explicit power analysis was used, you should describe how you decided what sample (replicate) size (number) to use

Please outline where this information can be found within the submission (e.g., page numbers or figure legends), or explain why this information doesn’t apply to your submission:

Power analysis is appropriate when effect size could be predicted. This is not the case in our studies, so power analysis is not applicable.

**Replicates**

- You should report how often each experiment was performed
- You should include a definition of biological versus technical replication
- The data obtained should be provided and sufficient information should be provided to indicate the number of independent biological and/or technical replicates
- If you encountered any outliers, you should describe how these were handled
- Criteria for exclusion/inclusion of data should be clearly stated
- High-throughput sequence data should be uploaded before submission, with a private link for reviewers provided (these are available from both GEO and ArrayExpress)

Please outline where this information can be found within the submission (e.g., page numbers or figure legends), or explain why this information doesn’t apply to your submission:

1. Clarification that N numbers reflect biological replicates is on Page 4 Line 64.

2. N numbers are included on Page 4 Line 64, Page 7 Line 144, Page 5 Line 159,

Page 8 Line 166, and Page 8 Line 172, and Page 10 Line 223, Page 12 Line 256, Page 12 Line 267. N Numbers are also included in all the figure captions, including both main and supplementary figure captions.

3. All column graphs and dot plots include raw data as well as error bars (S.E.M)

4. Main text time courses show the mean, with dotted lines above and below the curve to represent SEM. Traces for all individual cells, as well as average curve and S.E.M. for all time points are plotted in the supplemental figures. Summary dot plots showing magnitude of change in response are shown for each experiment, with the mean and SEM shown.

**Statistical reporting**

- Statistical analysis methods should be described and justified
- Raw data should be presented in figures whenever informative to do so (typically when N per group is less than 10)
- For each experiment, you should identify the statistical tests used, exact values of N, definitions of center, methods of multiple test correction, and dispersion and precision measures (e.g., mean, median, SD, SEM, confidence intervals; and, for the major substantive results, a measure of effect size (e.g., Pearson's r, Cohen's d)
- Report exact p-values wherever possible alongside the summary statistics and 95% confidence intervals. These should be reported for all key questions and not only when the p-value is less than 0.05.

Two-tailed paired t-test was used for comparing anisotropy before and after

stimulation (e.g. page 4 line 65). Two-tailed t-tests were also included to compare wild type and mutant variants for Venus-cp172Venus FLARE AKAR (Figure 1 legend), Venus-cp172Venus FLARE EKAR, and Venus-cp172Venus FLARE CKAR, as well as comparing KCl treated and vehicle-only control for FLARE MLCK (Figure 2 legend). Two-tailed t-test was also used to compare anisotropy before and after electrical stimulation and isoproterenol injection the in vivo anisotropy experiment (Figure 4 legend). For the D1ER sensor, because multiple conditions were being compared, an ANOVA and Multiple Tukey comparison test was used (see figure legend for Figure 3 Supplement 4).

Please outline where this information can be found within the submission (e.g., page numbers or figure legends), or explain why this information doesn’t apply to your submission:

(For large datasets, or papers with a very large number of statistical tests, you may upload a single table file with tests, Ns, etc., with reference to page numbers in the manuscript.)

**Additional data files (“source data”)**

- We encourage you to upload relevant additional data files, such as numerical data that are represented as a graph in a figure, or as a summary table
- Where provided, these should be in the most useful format, and they can be uploaded as “Source data” files linked to a main figure or table
- Include model definition files including the full list of parameters used
- Include code used for data analysis (e.g., R, MatLab)
- Avoid stating that data files are “available upon request”

Please indicate the figures or tables for which source data files have been provided:

Data for all graphs in Figures 1 through 4 are uploaded (e.g. “source_data_figure1.xlsx”)
